# Adolescents with chronic headaches- mental health problems and coping patterns

**DOI:** 10.1186/1129-2377-14-S1-P8

**Published:** 2013-02-21

**Authors:** S Hartberg, C Lundqvist, J Clench-Aas, RK Raanaas

**Affiliations:** 1 Norwegian University of Life Sciences, Norway; 2 HØKH, Research Centre, Akershus University Hospital, Norway; 3 Norwegian Institute of Public Health, Norway

## Introduction/purpose

Most studies on chronic headache have focused on adults, but chronic headache is also a major problem in children and adolescents [1]. There are gaps in knowledge of coping and mental health problems in adolescents with chronic headaches [2]. The aim of the present study is to get a better understanding of the relationship between different coping strategies and the presence of chronic headache either alone or in combination with mental health problems.

## Methods

This study is based on a self-report cross-sectional study undertaken in Akershus County in Norway in 2002. A total of 19,985 adolescents were included in this study, covering lower secondary and upper secondary students, aged 13-19 years. Mental health was assessed by using the Strengths and Difficulties Questionnaire (SDQ). Chronic headache was measured with a single item, defined in close accordance with the classification of the International Headache Society (ICHD-2). Internal and external coping strategies were assessed through seven items, based on the question: What do you do/what happens when you are burdened by painful thoughts and feelings?

## Results

Adolescents with chronic headaches showed more symptoms of mental health problems overall compared to those without chronic headache or with mental health problems alone. Logistic regression analyses showed that those adolescents having both chronic headaches and comorbid mental health problems to a greater extent used internal coping strategies, such as keeping feelings inside (OR 2.05), using drugs (OR 1.79) and talking oneself out of problems (OR 1.55), compared to those with chronic headache alone.

## Conclusion

We suggest that attention should be paid towards coping strategies used by a high risk group that have both chronic headaches and mental health problems.
